# *CASP8* -652 6N insertion/deletion polymorphism and overall cancer risk: evidence from 49 studies

**DOI:** 10.18632/oncotarget.18187

**Published:** 2017-05-25

**Authors:** Jiarong Cai, Qingjian Ye, Suling Luo, Ze Zhuang, Kui He, Zhen-Jian Zhuo, Xiaochun Wan, Juan Cheng

**Affiliations:** ^1^ Department of Urology, The Third Affiliated Hospital of Sun Yat-Sen University, Guangzhou 510630, China; ^2^ Department of Gynecology, The Third Affiliated Hospital of Sun Yat-Sen University, Guangzhou 510630, China; ^3^ Department of Otolaryngology, The First People's Hospital of Foshan (Affiliated Foshan Hospital of Sun Yat-Sen University), Foshan 528000, China; ^4^ Department of Joint Surgery and Orthopaedic Trauma, The Third Affiliated Hospital of Sun Yat-Sen University, Guangzhou 510630, China; ^5^ The Second People's Hospital of FuTian District, Shenzhen 518000, China; ^6^ School of Chinese Medicine, Faculty of Medicine, The Chinese University of Hong Kong, Hong Kong 999077, China; ^7^ Department of Pathology, Fudan University Shanghai Cancer Center, Shanghai 200032, China; ^8^ Department of Oncology, Shanghai Medical College, Fudan University, Shanghai 200032, China

**Keywords:** CASP8, -652 6N insertion/deletion, polymorphism, cancer risk, meta-analysis

## Abstract

The *CASP8* -652 6N insertion/deletion (I/D) polymorphism reduces expression of caspase 8. We conducted a meta-analysis to clarify the relationship between this polymorphism and cancer risk. Eligible articles were retrieved from PubMed, EMBASE, CNKI, and WANFANG databases through February 2017. A total of 33 articles with 49 studies, including 33,494 cases and 36,397 controls, were analyzed. We found that the *CASP8* -652 6N ins/del polymorphism was associated with decreased overall cancer risk in five genetic models [DD vs. II: odds ratio (OR)=0.76, 95% confidence interval (CI)=0.69–0.84, ID vs. II: OR=0.87, 95% CI=0.83–0.92, DD vs. ID/II: OR=0.82, 95% CI=0.75–0.89, ID/DD vs. II: OR=0.85, 95% CI=0.80–0.90, and D vs. I: OR=0.87, 95% CI=0.83–0.91]. Stratified analyses showed that the polymorphism was associated with decreased risk of colorectal, breast, esophageal, renal cell, lung, cervical, bladder, gastric, and other cancers. Overall cancer risk was reduced in Asian and Caucasian patients, both hospital- and population-based studies, and both high and low quality studies. Our results highlight the role of the *CASP8* -652 6N ins/del polymorphism in decreasing cancer risk. Further studies with large-cohort populations, especially for specific cancer types and ethnic groups, are needed to confirm our findings.

## INTRODUCTION

Cancer is a substantial public health burden worldwide and is the second leading cause of death in the United States. An estimated 1,688,780 new cancer cases and 600,920 cancer deaths will occur in the United States this year [1]. Approximately 14 million new cancer cases occurred worldwide in 2012, and by 2025, global cancer incidence is predicted to rise to 20 million new cases annually [2]. Although there are many cancer risk factors, genetic abnormalities play crucial roles in carcinogenesis [3–6].

Apoptosis is a control mechanism to prevent over-proliferation in normal cells [7], and apoptosis pathway aberrations are implicated in cancer development [8]. Caspases are the main regulatory enzymes in the apoptosis pathway [9]. Caspase 8 mediates the extrinsic apoptosis pathway [10, 11]. Human *CASP8* is located on chromosome 2q33∼q34, has 11 exons [12], and is highly polymorphic with more than 474 single nucleotide polymorphisms (SNPs) according to the dbSNP database (http://www.ncbi.nlm.nih.gov/SNP). The *CASP8* -652 6N ins/del polymorphism (rs3834129) is a six-nucleotide insertion/deletion variant located in the *CASP8* promoter region [13], and leads to decreased *CASP8* expression. Impaired caspase 8 function reduces T lymphocyte “activation-induced cell death” (AICD) activity, which is important in immune surveillance of cancer cells [13].

Extensive epidemiological studies have assessed the association between the *CASP8* -652 6N ins/del polymorphism and cancer risk. However, these studies have not produced conclusive results. The most recent previous meta-analysis of this association, conducted in 2014, assessed a relatively small number of studies. We performed this meta-analysis with a larger sample size to more precisely describe the association of interest.

## RESULTS

### Study characteristics

**Figure 1:**
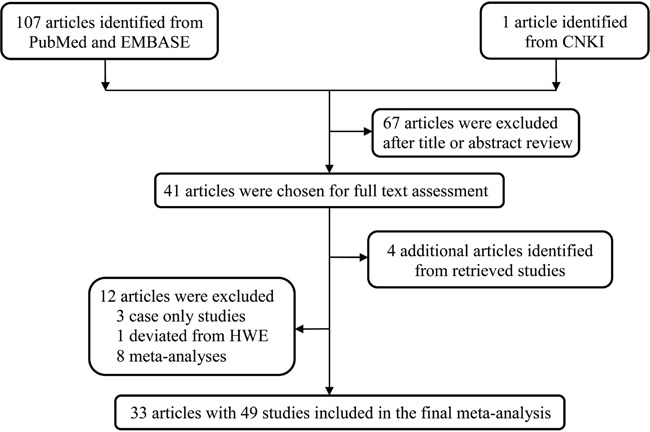
Flow diagram of the study selection process

Our study selection workflow is shown in Figure 1. Our systematic computer-based search initially identified 108 potentially relevant articles. After scanning titles and abstracts, 67 articles about unrelated topics were excluded. We further excluded 12 articles: eight were meta-analyses [14–21], three were case only studies [22–24], and one deviated from HWE [25]. Articles incorporating several ethnic groups or cancer types were separated into corresponding independent studies. In total, our analysis included datasets from 33 articles with 49 studies [13, 26–57].

Characteristics for 33,494 cases and 36,397 controls are summarized in Table 1. Of the included studies, 12 were conducted on colorectal cancer, nine on other cancers, eight on breast cancer, three on esophageal cancer, three on renal cell carcinoma, and two on lung cancer, cervical cancer, prostate cancer, bladder cancer, lymphoma cancer, and gastric cancer, respectively. Twenty-seven studies were conducted in Asians, 20 in Caucasians, one in Africans, and one in mixed populations. Twenty-four studies were of population-based design, 22 studies were of hospital-based design, and three did not mention study design in the original data. We also classified the studies as either low quality (25 studies) or high quality (24 studies) by quality score.

**Table 1:** Characteristics of studies included in the meta-analysis

Author last name	Year	Cancer type	Country	Ethnicity	Design	Genotype method	Case				Control				MAF	HWE	Score
							II	ID	DD	All	II	ID	DD	All			
Sun	2007	Lung	China	Asian	PB	PCR-RFLP	756	348	45	1149	640	407	64	1111	0.24	0.947	11
Sun	2007	Esophagus	China	Asian	PB	PCR-RFLP	652	328	38	1018	543	338	56	937	0.24	0.724	11
Sun	2007	Gastric	China	Asian	PB	PCR-RFLP	262	142	16	420	233	152	25	410	0.25	0.975	11
Sun	2007	Colorectal	China	Asian	PB	PCR-RFLP	605	280	33	918	528	304	58	890	0.24	0.116	11
Sun	2007	Breast	China	Asian	PB	PCR-RFLP	699	371	49	1119	513	419	72	1004	0.28	0.279	11
Sun	2007	Cervical	China	Asian	PB	PCR-RFLP	199	102	13	314	314	211	42	567	0.26	0.428	10
Yang	2008	Pancreatic	China	Asian	PB	PCR-RFLP	268	111	18	397	521	323	63	907	0.25	0.185	13
Pittman	2008	Colorectal	England	Caucasian	PB	AS-PCR	995	1897	987	3879	892	1872	897	3661	0.50	0.170	9
Frank	2008	Breast	Germany	Caucasian	HB	Fluorescent	298	535	221	1054	270	506	263	1039	0.50	0.403	7
Frank	2008	Breast	England	Caucasian	PB	Fluorescent	235	541	251	1027	245	608	321	1174	0.53	0.169	10
Frank	2008	Breast	Germany	Caucasian	PB	Fluorescent	280	509	222	1011	285	492	229	1006	0.47	0.550	9
Frank	2008	Breast	England	Caucasian	PB	Fluorescent	1133	2115	1050	4298	1149	2263	1062	4474	0.49	0.422	8
Cybulski	2008	Breast	Poland	Caucasian	PB	AS-PCR	178	314	126	618	274	499	192	965	0.46	0.195	6
Cybulski	2008	Prostate	Poland	Caucasian	PB	AS-PCR	139	236	110	485	274	499	192	965	0.46	0.195	6
Li	2008	Melanoma	USA	Caucasian	HB	PCR	243	385	177	805	207	440	188	835	0.49	0.116	11
Wang	2009	Bladder	China	Asian	HB	PCR-RFLP	238	115	12	365	205	138	25	368	0.26	0.786	10
Gangwar	2009	Bladder	India	Asian	HB	PCR-RFLP	121	84	7	212	133	101	16	250	0.27	0.584	9
De Vecchi	2009	Breast	Italy	Caucasian	PB	PCR-RFLP	162	301	117	580	106	206	94	406	0.49	0.752	7
Zhu	2010	RCC	China	Asian	HB	PCR-RFLP	226	119	8	353	205	139	21	365	0.25	0.686	11
Srivastava	2010	Gallbladder	India	Asian	PB	PCR-RFLP	147	69	12	228	122	84	24	230	0.29	0.103	11
Liu	2010	Colorectal	China	Asian	PB	PCR-RFLP	233	116	21	370	528	278	32	838	0.20	0.538	13
Li	2010	HNSCC	USA	Caucasian	HB	PCR–RFLP	311	456	256	1023	257	542	253	1052	0.50	0.324	10
Xiao	2011	Lymphoma	China	Asian	NM	PCR-PAGE	43	17	4	64	89	38	6	133	0.19	0.460	3
Xiao	2011	Lymphoma	China	Asian	NM	PCR-PAGE	49	23	3	75	63	40	4	107	0.22	0.442	3
Umar	2011	Esophageal	India	Asian	PB	PCR	139	103	17	259	138	93	28	259	0.29	0.046	11
Theodoropoulos	2011	Colorectal	Greece	Caucasian	HB	RFLP-PCR	103	201	98	402	120	254	106	480	0.49	0.194	9
Malik	2011	Esophageal	India	Asian	HB	RFLP-PCR	68	59	8	135	96	75	24	195	0.32	0.127	8
Malik	2011	Gastric	India	Asian	HB	RFLP-PCR	59	44	5	108	96	75	24	195	0.32	0.127	8
Ma	2011	Ovarian	China	Asian	HB	MassARRAY	128	87	3	218	138	122	25	285	0.30	0.789	8
Liamarkopoulos	2011	Gastric	Greece	Caucasian	HB	PCR-RFLP	35	42	11	88	120	254	106	480	0.49	0.194	7
Hart	2011	Lung	Norway	Caucasian	PB	TaqMan	125	210	101	436	106	209	118	433	0.51	0.481	10
Chatterjee	2011	Cervical	South Africa	African	HB	PCR-RFLP	18	63	25	106	43	129	85	257	0.58	0.614	6
Fu	2011	Prostate	China	Asian	HB	PCR-RFLP	257	132	17	406	211	159	38	408	0.29	0.315	10
Wang	2012	RCC	China	Asian	HB	PCR-RFLP	192	101	7	300	168	114	18	300	0.25	0.817	10
Wang	2012	PTC	China	Asian	HB	PCR–RFLP	65	45	8	118	106	92	15	213	0.29	0.408	7
Tong	2012	ALL	China	Asian	HB	PCR-RFLP	217	113	31	361	338	153	28	519	0.20	0.057	10
Hashemi	2012	Breast	Iran	Asian	HB	AS-PCR	113	107	16	236	79	91	33	203	0.39	0.434	6
George	2012	Prostate	India	Asian	HB	PCR-RFLP	84	69	12	165	116	83	6	205	0.23	0.050	9
Xiao	2013	Colorectal	China	Asian	HB	PCR-PAGE	187	107	11	305	212	115	15	342	0.21	0.905	7
Wu	2013	Colorectal	China	Asian	HB	PCR-SSCP	284	152	15	451	358	244	29	631	0.24	0.119	11
De Martino	2013	RCC	Austria	Caucasian	HB	PCR-RFLP	72	138	40	250	53	129	68	250	0.53	0.572	9
Pardini	2014	Colorectal	Spain	Caucasian	PB	Taqman	500	996	482	1978	425	802	420	1647	0.50	0.290	11
Pardini	2014	Colorectal	Italy	Caucasian	PB	Taqman	195	285	137	617	783	1230	538	2551	0.45	0.178	9
Pardini	2014	Colorectal	USA	Caucasian	PB	Taqman	237	514	259	1010	383	794	403	1580	0.51	0.835	9
Pardini	2014	Colorectal	England	Caucasian	PB	Taqman	410	825	341	1576	165	393	209	767	0.53	0.436	11
Pardini	2014	Colorectal	Czech	Caucasian	PB	Taqman	239	479	249	967	169	326	177	672	0.51	0.443	10
Pardini	2014	Colorectal	Netherlands	Caucasian	PB	Taqman	169	282	134	585	106	177	76	359	0.46	0.895	8
Tang	2015	OSCC	China	Asian	HB	PCR-RFLP	328	159	18	505	276	197	34	507	0.26	0.885	10
Carvalho	2015	ALL	Brazil	Mixed	NM	PCR	23	81	26	130	47	53	25	125	0.41	0.163	4

MAF: minor allele frequency; HWE: Hardy-Weinberg equilibrium; OSCC: oral squamous cell carcinoma; PTC: papillary thyroid carcinoma; RCC: renal cell carcinoma; HNSCC: head and neck squamous cell carcinoma; ALL: acute lymphocytic leukemia; PB: population based; HB: hospital based; NM: not mentioned; PCR-PAGE: polymerase chain reaction-polyacrylamide gel electrophoresis; PCR-RFLP: polymerase chain reaction-restriction fragment length polymorphism; AS-PCR: allele-specific polymerase chain reaction.

### Quantitative analysis

**Figure 2:**
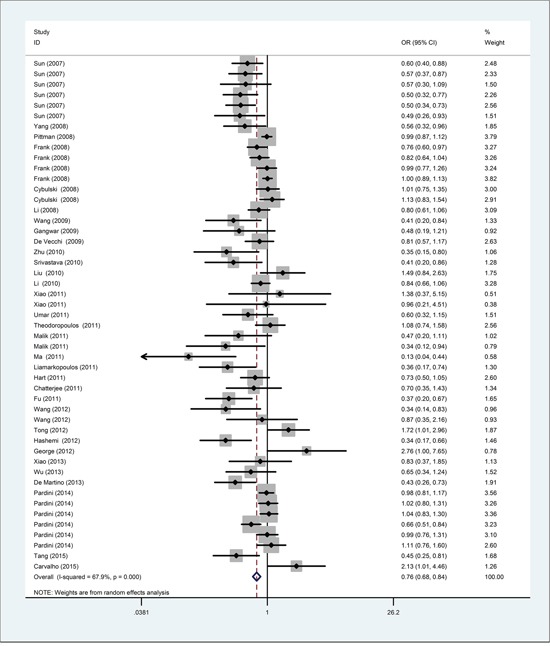
Forest plot of the association between the *CASP8* -652 6N ins/del polymorphism and cancer risk via the homozygous model The OR and 95% CI for each study are plotted as a box and horizontal line. ◊, pooled ORs and the corresponding 95% CIs.

Overall meta-analysis information is shown in Table 2 and Figure 2. In the pooled analysis, the *CASP8* -652 6N ins/del polymorphism was associated with reduced overall cancer risk in all five genetic models (homozygous: DD vs. II: odds ratio (OR)=0.76, 95% confidence interval (CI)=0.69–0.84; heterozygous: ID vs. II: OR=0.87, 95% CI=0.83–0.92; recessive: DD vs. ID/II: OR=0.82, 95% CI=0.75–0.89; dominant: ID/DD vs. II: OR=0.85, 95% CI=0.80–0.90; and allele: D vs. I: OR=0.87, 95% CI=0.83–0.91.

**Table 2:** Meta-analysis of the association between the *CASP8* -652 6N ins/del polymorphism and overall cancer risk

Variables	No. of studies	Sample size	Homozygous		Heterozygous		Recessive		Dominant		Allele	
			DD vs. II		ID vs. II		DD vs. ID/II		ID/DD vs. II		D vs. I	
			OR (95% CI)	P ^het^	OR (95% CI)	P ^het^	OR (95% CI)	P ^het^	OR (95% CI)	P ^het^	OR (95% CI)	P ^het^
All	49	33494/36397	**0.76 (0.69-0.84)**	<0.001	**0.87 (0.83-0.92)**	<0.001	**0.82 (0.75-0.89)**	<0.001	**0.85 (0.80-0.90)**	<0.001	**0.87 (0.83-0.91)**	<0.001
Cancer type												
Colorectal	12	13058/14418	0.93 (0.82-1.05)	0.018	**0.94 (0.88-0.99)**	0.529	0.96 (0.87-1.06)	0.019	**0.93 (0.87-1.00)**	0.190	0.96 (0.90-1.01)	0.012
Breast	8	9943/10271	**0.80 (0.67-0.96)**	0.001	**0.90 (0.81-1.01)**	0.018	**0.85 (0.74-0.99)**	0.002	**0.87 (0.77-0.99)**	0.002	**0.89 (0.80-0.98)**	<0.001
Esophageal	3	1412/1196	**0.56 (0.40-0.78)**	0.901	0.93 (0.74-1.17)	0.206	**0.58 (0.42-0.79)**	0.812	**0.83 (0.71-0.97)**	0.385	**0.81 (0.72-0.92)**	0.712
RCC	3	903/915	**0.39 (0.26-0.59)**	0.852	**0.78 (0.64-0.95)**	0.998	**0.46 (0.32-0.66)**	0.732	**0.71 (0.58-0.86)**	0.949	**0.70 (0.61-0.82)**	0.966
Lung	2	1585/1544	**0.66 (0.51-0.87)**	0.473	**0.75 (0.64-0.88)**	0.385	**0.75 (0.59-0.95)**	0.458	**0.73 (0.63-0.85)**	0.453	**0.78 (0.69-0.87)**	0.273
Cervical	2	420/824	**0.58 (0.36-0.93)**	0.456	0.86 (0.59-1.25)	0.230	**0.59 (0.39-0.88)**	0.728	**0.76 (0.59-0.98)**	0.355	**0.76 (0.63-0.92)**	0.556
Prostate	2	650/205	1.54 (0.67-3.55)	0.100	0.99 (0.79-1.23)	0.411	1.50 (0.74-3.07)	0.135	1.05 (0.85-1.29)	0.321	1.11 (0.93-1.33)	0.255
Bladder	2	577/618	**0.44 (0.25-0.77)**	0.799	0.79 (0.62-1.01)	0.334	**0.48 (0.27-0.84)**	0.907	**0.74 (0.59-0.93)**	0.317	**0.74 (0.61-0.90)**	0.338
Lymphoma	2	139/240	1.19 (0.44-3.23)	0.729	0.82 (0.52-1.31)	0.635	1.26 (0.47-3.39)	0.789	0.86 (0.56-1.34)	0.559	0.93 (0.64-1.35)	0.535
Gastric	2	196/675	**0.35 (0.19-0.63)**	0.939	0.74 (0.44-1.23)	0.145	**0.45 (0.26-0.78)**	0.538	0.64 (0.40-1.01)	0.171	**0.66 (0.51-0.84)**	0.487
ALL	2	491/644	**1.85 (1.20-2.87)**	0.655	1.83 (0.69-4.85)	0.004	1.32 (0.81-2.14)	0.228	1.79 (0.81-3.97)	0.014	**1.33 (1.10-1.61)**	0.443
Others	9	4120/4847	**0.57 (0.43-0.75**)	0.009	**0.72 (0.65-0.79)**	0.976	**0.65 (0.49-0.88)**	0.001	**0.70 (0.64-0.77)**	0.855	**0.75 (0.68-0.84)**	0.013
Ethnicity												
Asian	27	10569/11219	**0.58 (0.48-0.70)**	<0.001	**0.80 (0.75-0.85)**	0.231	**0.62 (0.52-0.74)**	0.002	**0.77 (0.72-0.83)**	0.016	**0.79 (0.73-0.84)**	<0.001
Caucasian	20	22689/24796	**0.90 (0.83-0.98)**	0.006	**0.92 (0.88-0.97)**	0.225	0.95 (0.89-1.02)	0.007	**0.92 (0.87-0.97**)	0.079	**0.95 (0.91-0.99)**	0.008
African	1	106/257	0.70 (0.35-1.43)	/	1.17 (0.62-2.19)	/	0.63 (0.37-1.05)	/	0.98 (0.54-1.80)	/	0.82 (0.60-1.13)	/
Mixed	1	130/125	**2.13 (1.01-4.46)**	/	**3.12 (1.70-5.73)**	/	1.00 (0.54-1.85)	/	**2.80 (1.57-5.00)**	/	**1.50 (1.05-2.12)**	/
Source of control												
PB	24	25259/26848	**0.83 (0.75-0.92)**	<0.001	**0.89 (0.84-0.94)**	0.008	**0.89 (0.82-0.96)**	<0.001	**0.87 (0.81-0.93)**	<0.001	**0.89 (0.85-0.95)**	<0.001
HB	22	7966/9184	**0.61 (0.49-0.75)**	<0.001	**0.83 (0.77-0.89)**	0.213	**0.67 (0.55-0.82)**	<0.001	**0.79 (0.73-0.87)**	0.024	**0.81 (0.75-0.88)**	<0.001
NM	3	269/365	1.73 (0.95-3.14)	0.619	1.30 (0.53-3.20)	0.003	1.07 (0.63-1.80)	0.896	1.29 (0.58-2.88)	0.005	1.14 (0.79-1.64)	0.156
Quality score												
>9	24	16745/16831	**0.67 (0.58-0.77)**	<0.001	**0.81 (0.76-0.87)**	0.008	**0.75 (0.66-0.85)**	<0.001	**0.78 (0.73-0.84)**	<0.001	**0.81 (0.76-0.87)**	<0.001
≤9	25	16749/19566	**0.87 (0.77-0.99)**	<0.001	0.95 (0.90-1.01)	0.289	**0.90 (0.81-1.00)**	<0.001	**0.94 (0.88-1.00)**	0.048	**0.94 (0.90-0.99)**	<0.001

Values were in bold, if the 95% CI excluded 1 or *P*<0.05.

Het: heterogeneity; RCC: renal cell carcinoma; ALL: acute lymphocytic leukemia; HB: hospital based; PB: population based; NM: not mentioned.

In cancer type stratification analysis, the *CASP8* -652 6N ins/del polymorphism decreased risk for colorectal cancer, breast cancer, esophageal cancer, renal cell carcinoma, lung cancer, cervical cancer, bladder cancer, gastric cancer, and other cancers. However, acute lymphocytic leukemia risk was increased (DD vs. II: OR=1.85, 95% CI=1.20–2.87; and D vs. I: OR=1.33, 95% CI=1.10–1.61). We observed no correlations between the *CASP8* -652 6N ins/del polymorphism and prostate cancer or lymphoma.

Stratification analysis by ethnicity revealed a decreased cancer risk for Asians (DD vs. II: OR=0.58, 95% CI=0.48–0.70) and Caucasians (DD vs. II: OR=0.90, 95% CI=0.83–0.98), and an increased risk in mixed populations (DD vs. II: OR=2.13, 95% CI=1.01–4.46). We also found that the *CASP8* -652 6N ins/del polymorphism decreased cancer risk in population-based (DD vs. II: OR=0.83, 95% CI=0.75–0.92) and hospital-based groups (DD vs. II: OR=0.61, 95% CI=0.49–0.75). Similarly, the *CASP8* -652 6N ins/del polymorphism was associated with decreased cancer risk in both the high quality (DD vs. II: OR=0.67, 95% CI=0.58–0.77) and low quality study groups (DD vs. II: OR=0.87, 95% CI=0.77–0.99).

### Heterogeneity and sensitivity analysis

Heterogeneity was observed in all five genetic models (*P*<0.001, Q test). Therefore, the random-effect model was adopted to generate ORs and 95% CIs. We also conducted a sequential leave-one-out sensitivity analysis to evaluate the impact of a single study on the pooled estimates. Omission of no single study influenced the pooled ORs, indicating the statistical robustness of this meta-analysis (data not shown).

### Publication bias

**Figure 3:**
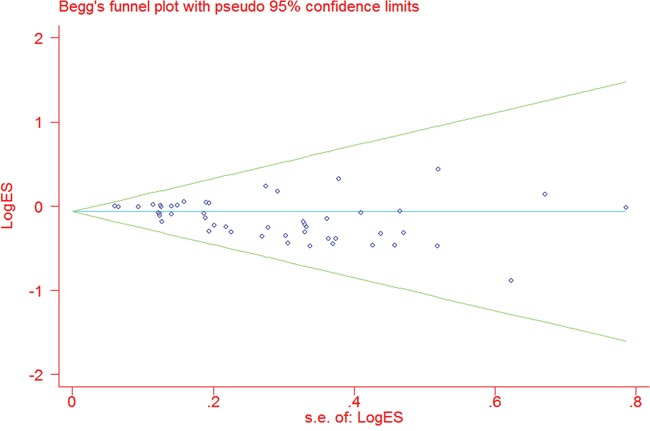
Funnel plot analysis to detect publication bias for the *CASP8* -652 6N ins/del polymorphism via the homozygous model Each point represents a separate study for the indicated association.

Begg's funnel plot shapes did not suggest any obvious asymmetry (Figure 3). Egger's test results (DD vs. II: t=-4.17, *P*<0.001; ID vs. II: t=-0.12, *P*=0.905; DD vs. ID/II: t=-1.15, *P*=0.257; ID/DD vs. II: t=-1.09, *P*=0.281; and D vs. I: t=-3.33, *P*=0.002) suggested that publication bias existed in the homozygote and allele models.

### Trial sequential analysis

**Figure 4:**
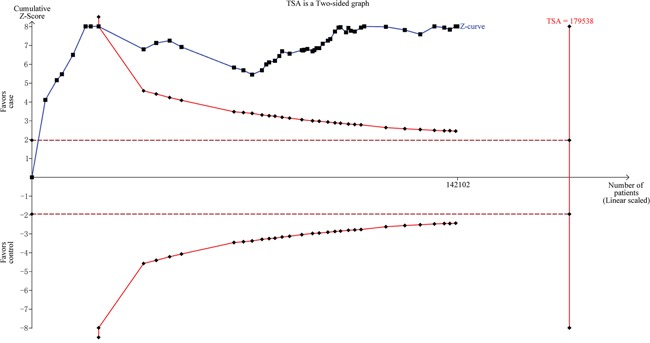
Trial sequential analysis for the *CASP8* -652 6N ins/del polymorphism via the allele contrast model

To minimize random errors and strengthen the robustness of our conclusions, we performed trial sequential analysis (TSA) (Figure 4). The cumulative Z-curve crossed the trial sequential monitoring boundary before the required information size was reached, suggesting that our study conclusion was convincing and no additional evidence was needed to verify said conclusion.

## DISCUSSION

The present meta-analysis comprehensively evaluated the relationship between the *CASP8* -652 6N ins/del polymorphism and cancer risk across 49 studies (33,494 cases and 36,397 controls). The *CASP8* -652 6N ins/del polymorphism was associated with decreased cancer risk in all five genetic models, and in the following subgroups: colorectal cancer, breast cancer, esophageal cancer, renal cell carcinoma, lung cancer, cervical cancer, bladder cancer, gastric cancer, other cancers, Asian, Caucasian, mixed population, population-based controls, hospital-based controls, high quality score, and low quality score.

Human immune cells play critical roles in eliminating potentially malignant cells [58]. Caspase 8 protein (encoded by *CASP8*) maintains immune cells by mediating the activation-apoptosis balance [59]. Low caspase 8 expression or functional aberrations may decrease T lymphocyte apoptotic reactivity [13]. The *CASP8* -652 6N del variant inactivates the transcription factor stimulatory protein 1 binding site, decreasing *CASP8* transcription [13]. Thus, this variant may affect cancer susceptibility by influencing immune surveillance.

The first case-control study of the *CASP8* -652 6N del variant-cancer association, with 4,995 cases and 4,972 controls, was conducted by Sun, *et al*. in 2007 [13]. The authors found that the *CASP8* -652 6N deletion allele decreased susceptibility to lung, colorectal, esophageal, breast, cervical, and gastric cancers. Biochemical assays illustrated that this variant might decrease apoptotic reactivity in cancer cell-stimulated T lymphocytes. However, Umar, *et al*. did not detect any association between the *CASP8* -652 6N polymorphism and esophageal squamous cell carcinoma (ESCC) risk in 259 patients and 259 healthy controls in an Indian population [45]. Several meta-analyses have attempted to address these contradictory conclusions. A 2012 meta-analysis by Chen, *et al*., including 19 case-control studies with 23,172 cases and 26,532 controls, associated the del allele, ins/del genotype, and del allele carriers with reduced overall cancer risk [16]. Similarly, in a meta-analysis incorporating 11 reports with 27,459 cases and 31,614 controls, Yin, *et al*. associated the *CASP8* -652 5N del polymorphism with reduced overall cancer risk via homozygous, dominant, and recessive models [15]. In 2014, breast cancer- and colorectal cancer-specific meta-analyses [19, 20] concluded that the *CASP8* -652 6N del polymorphism reduced cancer risk. However, no association was observed between this polymorphism and prostate cancer susceptibility in a meta-analysis by Zhang, *et al*. [21].

To provide a more robust clarification, our meta-analysis included all eligible studies published in either the English or Chinese language. In agreement with the four previously published meta-analyses, we found that the *CASP8* -652 6N ins/del polymorphism was associated with reduced overall cancer risk. In subgroup analyses, the polymorphism was associated with reduced risk of colorectal cancer, breast cancer, esophageal cancer, renal cell carcinoma, lung cancer, cervical cancer, bladder cancer, gastric cancer, and other cancers, but not prostate cancer or lymphoma. A prostate cancer-specific meta-analysis also failed to detect a significant association. This may be attributed to cancer-specific inherent heterogeneity [60, 61]. Additionally, we observed an association with decreased cancer risk among Asians and Caucasians, but not Africans or mixed ethnicity populations. However, the limited number of studies in Africans and mixed ethnicity population may account for this finding, and *CASP8* -652 6N ins/del polymorphism allelic distributions might vary geographically and ethnically.

Our meta-analysis of the association between the *CASP8* -652 6N ins/del polymorphism and cancer risk is by far the largest such meta-analysis with the greatest statistical power published thus far. We conducted subgroup analyses to provide a more precise, cancer type-specific conclusion, and we assessed studies in both Chinese and English to minimize selection bias. However, our study had certain limitations. First, for some types of cancers, the calculated association was not robust enough due to limited numbers of original studies. Second, only one *CASP8* genetic variant was considered, and confounding factors, such as other genetic mutations and environmental exposures, also influence cancer susceptibility. Third, the observed between-study heterogeneity may reduce the validity of our conclusions. Finally, publication bias, language bias, or selection bias might lead to false positive or negative findings.

The present work robustly concludes that the *CASP8* -652 6N ins/del polymorphism is associated with reduced overall cancer risk. Refined studies with larger sample sizes, especially for certain cancer types and ethnic groups, are needed to fully validate this relationship.

## MATERIALS AND METHODS

### Search strategy

We conducted a literature search in PubMed and EMBASE using the following combined terms: ‘*Caspase 8*’ or ‘*CASP8*’ and ‘polymorphism’ or ‘polymorphisms’ or ‘single nucleotide polymorphism’ or ‘SNP’ or ‘variant’ and ‘cancer’ or ‘tumor’ or ‘carcinoma’ or ‘carcinogenesis’ or ‘neoplasm’. We also searched studies written in Chinese from two databases, WANFANG and CNKI. We searched for articles published through February 2017 without imposing language limitations. Relevant references were also collected from retrieved articles. Only the largest or the most recent study was retained if studies contained overlapping data.

### Inclusion/exclusion criteria

Studies included in our analysis met the following criteria: (1) evaluated *CASP8* -652 6N ins/del polymorphism with respect to cancer risk; (2) case-control design; (3) sufficient information to extract genotype frequencies for all subjects; (4) genotype frequency of controls consistent with Hardy-Weinberg equilibrium (HWE); (5) publication language was English or Chinese. Criteria for exclusion included: (1) abstract only, review, or meta-analysis; (2) case only studies; (3) no detailed genotyping data provided; (4) repeated publication.

### Data extraction

Two authors (Jiarong Cai and Qingjian Ye) independently identified all eligible studies, and extracted data was included in the meta-analysis following consensus. The following items were recorded from each study: first author's name, year of publication, country, patient ethnicity, cancer type, source of controls, genotyping method, and genotype distributions of cases and controls. If reports contained more than one ethnic group or cancer type, we separated them into different studies.

### Trial sequential analysis

After adopting a risk of 5% for type I errors and 30% for type II errors, the required information size (sample sizes from all included trials) was calculated. TSA monitoring boundaries were built based on required information size and risk for type I and type II errors. If the cumulative Z-curve crossed the TSA monitoring boundary before the required information size was reached (i.e. if a sufficiently small *P*-value was achieved), further trials were unnecessary.

### Statistical analyses

We used the Chi-square test to ensure that all control genotype frequencies were in agreement with HWE. Odds ratios (ORs) with corresponding 95% confidence intervals (CIs) obtained from case and control genotype frequencies were used to assess the strength of association between the *CASP8* -652 6N ins/del polymorphism and cancer risk. Pooled ORs were calculated for the following five genetic models: homozygote model (DD vs. II), heterozygote model (ID vs. II), recessive model (DD vs. ID/II), dominant model (ID/DD vs. II), and allele model (D vs. I). The Cochran's Chi-square-based Q-test and the inconsistency index (I^2^ statistics) were adopted to assess heterogeneity between study results. I^2^<50% or *P*>0.10 indicates heterogeneity. The fixed-effects model (Mantel-Haenszel method) was used to estimate the pooled OR if no heterogeneity existed (I^2^<50% or *P*>0.10). Otherwise, the random-effects model (DerSimonian and Laird method) was applied. Quality assessment for each study was performed using the quality assessment criteria described previously (Supplementary Table 1) [62–65]. To decrease heterogeneity among studies, we conducted stratification analyses by ethnicity, cancer type, control source, and quality score. By adopting one-way sensitivity analysis, we recalculated the pooled ORs to assess the robustness of the results. We also conducted Begg's funnel plot and Egger's regression asymmetry test to examine potential publication bias [66–69]. STATA software v. 11.0 (Stata Corporation, College Station, TX) was used for statistical analyses [70]. *P*<0.05 (two-sided) was considered statistically significant.

## SUPPLEMENTARY MATERIALS TABLES


