# Supplementary material for: CASP8 -652 6N insertion/deletion polymorphism and overall cancer risk: evidence from 49 studies
Source: Oncotarget. 2017 May 25;8(34):56780–90. doi: 10.18632/oncotarget.18187 (PMC5593601; doi:10.18632/oncotarget.18187)
Supplement: Supplementary file 1 [file oncotarget-08-56780-s001.pdf]

CASP8 -652 6N insertion/deletion polymorphism and overall cancer risk: evidence from 49 studies

SUPPLEMENTARY MATERIAL

Supplementary Table 1: Score of quality assessment

Criteria	Score
Representativeness of cases	
Selected from population cancer registry	2
Selected from hospital	1
No method of selection described	0
Representativeness of controls	
Population-based	3
Blood donors	2
Hospital-based	1
Not described	0
Ascertainment of cancer cases	
Histopathologic confirmation	2
Patient medical record	1
Not described	0
Control selection	
Controls matched with cases by age and sex	2
Controls matched with cases only by age or by sex	1
Not matched or not descried	0
Genotyping examination	
Genotyping done blindly and quality control	2
Only genotyping done blindly or quality control	1
Unblinded and without quality control	0
Total sample size for both cases and controls	
Larger than 1000	3
Larger than 500, but less than 1000	2
Larger than 200, but less than 500	1
Less than 200	0
